# Metagenomics Analysis Reveals the Composition and Functional Differences of Fecal Microbiota in Wild, Farm, and Released Chinese Three-Keeled Pond Turtles (*Mauremys reevesii*)

**DOI:** 10.3390/ani14121750

**Published:** 2024-06-10

**Authors:** Ijaz Khan, Rongping Bu, Zeeshan Ali, Muhammad Shahid Iqbal, Haitao Shi, Li Ding, Meiling Hong

**Affiliations:** 1 Key Laboratory of Tropical Island Ecology, Ministry of Education, Hainan Key Laboratory of Tropical Animal and Plant Ecology, College of Life Sciences, Hainan Normal University, Haikou 571158, China; ijazkhan8486@gmail.com (I.K.); 18778395529@163.com (R.B.);; 2 College of Marine Science, Guangxi Key Laboratory of Beibu Gulf Biodiversity Conservation, Beibu Gulf University, Qinzhou 535000, China

**Keywords:** *Mauremys reevesii*, 16S rRNA gene sequencing, metagenomics analysis, habitat tracking, intestinal microbiota, conservation

## Abstract

**Simple Summary:**

The Chinese three-keeled pond turtle is an endangered species in the wild but with large-scale farming. However, it is difficult to distinguish individuals belonging to different habitats from their appearance. The current study investigates intestinal microbial diversity, metabolic pathways, and resistance to various types of antibiotics among wild, farm, and released turtles. The results showed that at the phylum level, Bacteroidota, Firmicutes, Fusobacteriota, and Actinobacteriota were abundant in the wild group, but Chloroflexi was only dominant in the farm and released groups. In addition, most genera increased in the groups of farm and released turtles, while few were abundant in the wild group. A metabolic pathway analysis revealed heightened activity in the associated pathways in the wild turtles. The antibiotic resistance gene *macB* was higher in the farm turtle group, while *tetA* was more abundant in the wild turtles, and *srpYmcr* was abundant in the released group. These outcomes were deemed helpful for a better understanding of the relationship between the intestinal microbiota of *Mauremys reevesii* and its habitats and could be useful for tracking habitats to protect, manage, and conserve this endangered species.

**Abstract:**

The intestine of living organisms harbors different microbiota associated with the biological functioning and health of the host and influences the process of ecological adaptation. Here, we studied the intestinal microbiota’s composition and functional differences using 16S rRNA and metagenomic analysis in the wild, farm, and released Chinese three-keeled pond turtle (*Mauremys reevesii*). At the phylum level, Bacteroidota dominated, followed by Firmicutes, Fusobacteriota, and Actinobacteriota in the wild group, but Chloroflexi was more abundant in the farm and released groups. Moreover, *Chryseobacterium*, *Acinetobacter*, *Comamonas*, *Sphingobacterium*, and *Rhodobacter* were abundant in the released and farm cohorts, respectively. *Cetobacterium*, *Paraclostridium*, *Lysobacter*, and *Leucobacter* showed an abundance in the wild group. The Kyoto Encyclopedia of Genes and Genomes (KEGG) database revealed that the relative abundance of most pathways was significantly higher in the wild turtles (carbohydrate metabolism, lipid metabolism, metabolism of cofactors, and vitamins). The comprehensive antibiotic resistance database (CARD) showed that the antibiotic resistance gene (ARG) subtype *macB* was the most abundant in the farm turtle group, while *tetA* was higher in the wild turtles, and *srpYmcr* was higher in the released group. Our findings shed light on the association between the intestinal microbiota of *M. reevesii* and its habitats and could be useful for tracking habitats to protect and conserve this endangered species.

## 1. Introduction

The intestine of living organisms harbors millions of microorganisms which jointly work as a “hidden organ” [1]. These intestinal microbiotas perform an important role in food degradation [2,3], nutrient supply [4], digestion and absorption [5], immune modulation [6], resistance to pathogen invasion [7], behavior [8], and emotional regulation [9] and are essential for ecological adaptation [3,10]. There are numerous factors that influence gut microbiota, including host diet, genetic and environmental history, physiological status, medication, stressors, and habitats [5]. Among these factors, diet and habituating environmental conditions are major factors that substantially influence the makeup of a microbial community [11]. Microbial imbalances could potentially lead to infection with opportunistic pathogens, inflammatory bowel disease, obesity, and autoimmune disorders [12,13,14,15]. The gut microbiota of invertebrates [16], amphibians [17], reptiles [18], and mammals [19] has been widely studied. A few studies have reported that diet, geography, ontogenesis [20], age, gender, and health status influence microbiota [21]; hence, this study explored the effect of habitat variation on intestinal microbiota in *Mauremys reevesii*.

In the current research, we focus on the freshwater *M. reevesii*, listed as an endangered species by the International Union for the Conservation of Nature (IUCN). In the past, it was distributed widely in China, part of the Korean Peninsula, and Japan [22], while currently, it is hard to find the individual in the field due to habitat destruction and overexploitation over the last couple of decades [23,24]. Therefore, this endangered species urgently requires conservation efforts. Habitat tracking has important implications for effective habitat planning, management, and the conservation of endangered species [25]. For this purpose, these turtles can be grown on turtle farms and then released into the wild, which could be a beneficial step in their conservation. Similarly, the comparative analysis of gut microbiota plays a crucial role in their preventive measures. Habitat has a direct influence on the gut microbiome, and their differences help in the tracking of host habitats [26]. For aquatic organisms with poor genetic markers requiring the identification of wild and farm inhabitants, the gut microbiome can serve as an alternate biomarker of habitat tracking [26,27]. Furthermore, the understanding of microbial taxonomic and functional variation may influence the host’s health. For this purpose, various techniques, such as 16S rRNA gene sequencing and metagenomic analysis, have advanced our understanding of the organism’s microbiome by allowing for the discovery and characterization of unculturable microbes with a prediction of their function [28]. Previously, 16S rRNA high-throughput sequencing technology has been widely applied to study the gut microbial structure and diversity of various endangered species [29,30]. The intestinal microbiota of wild-captured green sea turtles (*Chelonia mydas*) was dominated by Firmicutes, Bacteroidetes, and Proteobacteria, while the control group was dominated by Proteobacteria, Bacteroidetes, and Firmicutes, respectively [31]. Moreover, captive and wild oriental white storks (*Ciconia boyciana*) were dominated by Firmicutes, Actinobacteria, and Proteobacteria, while the highest diversity was confirmed in the wild group [32]. In addition, the captive tokay gecko (*Gekko gecko*) had significantly higher alpha diversity than wild populations, while both showed substantial differences in beta diversity [33]. Furthermore, Fong et al. (2020) found that Proteobacteria were abundant in the wild, while Firmicutes exhibited more abundance in captive beal’s eyed turtles (*Sacalia bealei*) at the phylum level. At the genus level, beal’s eyed turtles have a higher relative abundance of *Cetobacterium* and *Citrobacter* in the wild, whereas *Clostridium* species were much higher in captive turtles [20].

To date, various metagenomic analyses of intestinal microbiota have been performed with vertebrates, such as humans [34], cattle [35], red swamp fish (*Procambarus clarki*) [36], amphibious mudskippers (*Periophthalmus chrysospilos*) [37], chickens [38], pigs [39], amur tigers [40], and in between hawksbills (*Eretmochelys imbricata*) and green sea turtles (*Chelonia mydas*) [41]. In addition, a chicken multi-kingdom microbiome catalog has been studied, including bacterial, archaeal, and viral genomes, and this has provided functional insights into the chicken gut microbiota, paving the way for microbial interventions to improve chicken gut health and productivity [42]. Another study demonstrated that the gut microbiome of humans, chickens, and pigs contains genes for resistance to at least 20 antibiotics [43]. Metagenomic analysis helps researchers unveil the functional capacity of microbiomes related to metabolism and better understand and classify ARGs [36,44]. However, knowledge about the intestinal microbiota of *M. reevesii* is still limited. Therefore, the intestinal microbiota, including diversity and composition, metabolic pathways, and ARGs of wild, farm, and released *M. reevesii*, was investigated through a metagenomic sequencing analysis. Our results will aid in understanding the effects of various habitats on the composition, diversity, and functions of the intestinal microbiota, the identification of ARGs in *M. reevesii*, and the tracking of habitats that will be beneficial for the population conservation of this endangered species.

## 2. Materials and Methods

### 2.1. Fecal Sampling

We captured twenty-seven healthy adult male *M. reevesii* species from the wild and farms and categorized them into three groups (wild: *n* = 9; released: *n* = 9; and farm: *n* = 9) in Qichun County (29°590–30°400 N, 115°120–115°560 E) (Latitude: 30°27′8.39″ N; Longitude: 115°38′29.39″ E), Hubei Province, China. The wild and released turtles were randomly captured in the selected area, and we could distinguish between them on the basis of our knowledge of their growth behavior. Usually, individuals on farms grow faster before they are released, so the early copper rings of the carapace were wider, while the rings of the wild individuals were narrower. Actually, some people purchase the turtles from the farm and release them to the wild for some purpose. And most of these released individuals come from farms because farmed individuals are much cheaper than wild individuals. In addition, farm individuals were selected from a local turtle farm. The captured turtles in each group were individually kept in separate buckets until defecation. The fecal contents were accessed using sterilized forceps to reduce contamination as much as possible. The collected samples were immediately placed in 2 mL sterilized cryogenic vials, frozen using liquid nitrogen, transported to a laboratory on dry ice, and stored at −80 °C for the subsequent extraction of DNA. They were then further used for 16S rRNA gene sequencing and metagenomics analysis.

### 2.2. Bacterial DNA Extraction and Amplification

Total microbial DNA was extracted from the frozen fecal contents of nine individuals from each group (wild: *n* = 9; farm: *n* = 9; and released: *n* = 9) using the E.Z.N.A.™ Soil DNA Kit (D4015, Omega, Inc., Norwalk, CT, USA), according to the manufacturer’s instructions. The DNA quality and concentration were assessed using a NanoDrop 2000 spectrophotometer (Thermo Fisher Scientific; Wilmington, NC, USA), and 6 samples from wild turtles, 5 from farm turtles, and 5 from released individuals were selected for amplification. The hypervariable V3-V4 regions of the 16S rRNA gene were amplified using a PCR thermocycler system (GeneAmp 9700, ABI, Los Angeles, CA, USA) with the 338F (5′-ACTCCTACGGGAGGCAGCAG-3′) and 806R (5′-GGACTACHVGGGTWTCTAAT-3′) primers. PCR reactions for each sample were performed in triplicate in 20 μL reaction solutions containing 0.4 μL of 5× FastPfu Buffer, 2 μL of 2.5 mM dNTPs, 0.8 μL of each primer (5 μM), 0.4 μL of FastPfu polymerase, and 10 ng of template DNA. The following thermal cycler program was used for amplification: 3 min at 95 °C, 27 cycles of 30 s at 95 °C, 30 s at 55 °C, and 45 s at 72 °C, and a final extension at 72 °C for 10 min. The amplified product was sequenced after extraction from the gel using Illumina-based high-throughput sequencing from MajorBio Co., Ltd., Shanghai, China.

### 2.3. 16S rRNA Gene Sequencing Analysis

To analyze, quality screen, and merge raw fastq sequences, Trimmomatic and FLASH were used. Based on a 97% similarity cutoff, operational taxonomic units (OTUs) were clustered, and chimeric sequences were detected and eliminated using UCHIME [45]. For each library, the alpha diversity index reflects the community diversity (Shannon and Simpson) and community richness (Ace and Chao) [46]. Additionally, Mothur was used to generate a rarefaction curve and species accumulation plots for the confirmation of the sequencing depth [47]. The beta diversity was assessed by PCoA to screen OTUs based on unweighted UniFrac distances, and the difference was confirmed through the ANOSIM/Adonis test by using the R language vegan package [48].

### 2.4. Metagenomics Sequencing Analysis

To further analyze the functional capability of the microbiome, we randomly selected three fecal samples from each group (wild: *n* = 3; farm: *n* = 3; and released: *n* = 3) for metagenomic sequencing analysis on an Illumina sequencing HiSeq platform (Illumina; San Diego, CA, USA) according to the standard protocols of Majorbio Bio pharm Co., Ltd. (Shanghai, China). After the sequencing, raw reads were cleaned by Readfq (version 8), and the data were subjected to a BLAST search against the host database using Bowtie (version 2.2.4) to filter the reads. The metagenome was assembled using a combination of single assembly and mixed assembly. For each sample, SOAP denovo (version 2.04) was used to conduct single-sample assembly [49]. Clean data from all samples were compared to each scaffold by using Bowtie 2 software and obtaining unused paired-end reads. All samples were then combined, and SOAP denovo and MEGAHIT (version 1.04-beta) were used for mixed assembly. Fragments shorter than 500 bp in the scaftigs generated from the single or mixed assembly were filtered out for the statistical analysis. MetaGeneMark (version 2.10) [50] was then used to predict non-redundant (Nr) genes on scaftigs, and CD-HIT software (version 4.5.8) was employed to acquire the unique initial gene catalog [51]. Clean data for each sample were mapped to the initial gene catalog using Bowtie to obtain the number of reads and the statistical abundance of each gene in each sample. Moreover, the Nr genes were analyzed using BLAST against databases including KEGG, followed by DIAMOND, which provides information about metabolism, human diseases, etc. In addition, the comprehensive antibiotic resistance database (CARD) was used to align the unigenes annotated by using resistance gene identifier (version 5) software.

### 2.5. Statistical and Data Analysis

SPSS (v26) was employed to analyze the microbial communities, the abundance of ARGs of various kinds, and KEGG pathways among the different groups of *M. reevesii*. The data were subjected to the Kolmogorov–Smironov test and homogeneity variance test (K-S and H-V) to confirm normality and uniformity. Further, a one-way ANOVA test was used for the analysis of homogeneous and normally distributed data, and the Kruskal–Wallis H nonparametric test was used. The *p* values for microbial abundance, ARGs, and KEGG pathways (*p* < 0.05) were considered statistically significant. A Venn diagram, PCoA, and Adonist were carried out using the vegan package [52], and Circos (v0.69-3) was used to illustrate the abundance of functional genes [53]. Origin (8.5) was used to create stack columns and bar graphs.

## 3. Results

### 3.1. Intestinal Microbial Community Structure, Composition, and Diversity

Using a high-throughput 16S rRNA gene sequencing analysis for the three groups (wild: *n* = 6; farm: *n* = 5; and released: *n* = 5), we found a total of 617,408 raw reads with an average of 38,588 per sample, and the average length of each sequence was 418 bp (412–425). The curve analysis exhibited that the rarefaction observed per sample was adequate (Figure S1). Moreover, 335 OTUs were common among all three groups, while 34 OTUs (5.71% of all sequences) were common between the wild and released groups. In addition, 148 OTUs (24.87% of all the sequences in the farm turtles), three OTUs (OTU611, OTU672, and OTU1398), which consisted of 0.50% of all the sequences in the wild turtles, and one OTU175, which covered 0.17% of all the sequences in the released turtles, were unique (Figure S2). In addition, according to richness estimators (ACE and Chao1), the abundances of microbial species were significantly higher in the farm turtles as compared to the wild and released turtles (*p* < 0.05). Additionally, the Shannon and Simpson estimators, representing the diversity of microbial species, showed significant differences only between the farm and released turtles (Table 1).

**Figure 1:**
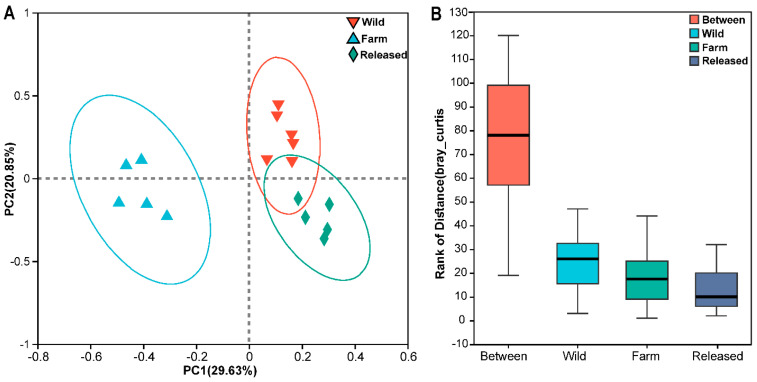
The clustering of intestinal microbiota in wild, farm, and released *M*. *reevesii*. (**A**) Principal coordinate (PCoA) analysis; (**B**) ANOSIM/Adonis test. The *X*-axis is the distance value among the groups, while the *Y*-axis represents the size of the distance value.

The PCoA of the unweighted UniFrac distance confirmed that the intestinal bacteria in all three *M. reevesii* groups were separate from each other (Figure 1A). The ANOSIM/Adonist test revealed different compositions among the groups (Figure 1B).

**Figure 2:**
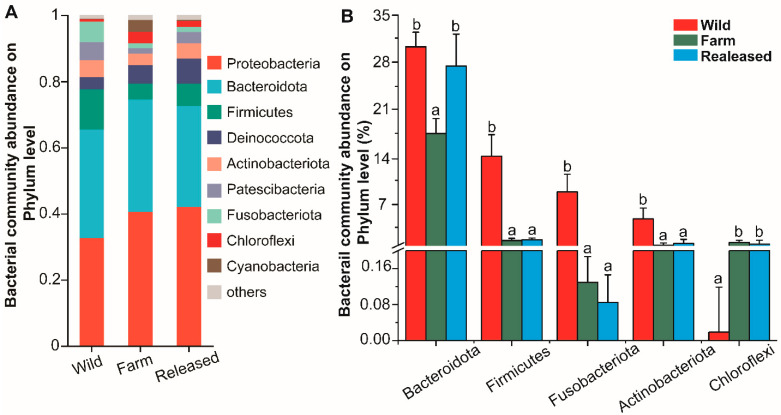
Comparison and relative abundances of intestinal bacterial phyla in wild, farm, and released *M*. *reevesii*. (**A**) The most dominant phyla. (**B**) The significant bacterial community abundance at the phylum level. The different lowercase letters represent significant differences between groups (*p* < 0.05).

At the phylum level, Bacteroidota (32.62%), Firmicutes (14.84%), Fusobacteriota (9.03), and Actinobacteriota (4.67%) were significantly abundant in the wild turtles (Figure 2). Meanwhile, Chloroflexi was more abundant in the farm (0.78%) and released (0.53%) groups.

**Figure 3:**
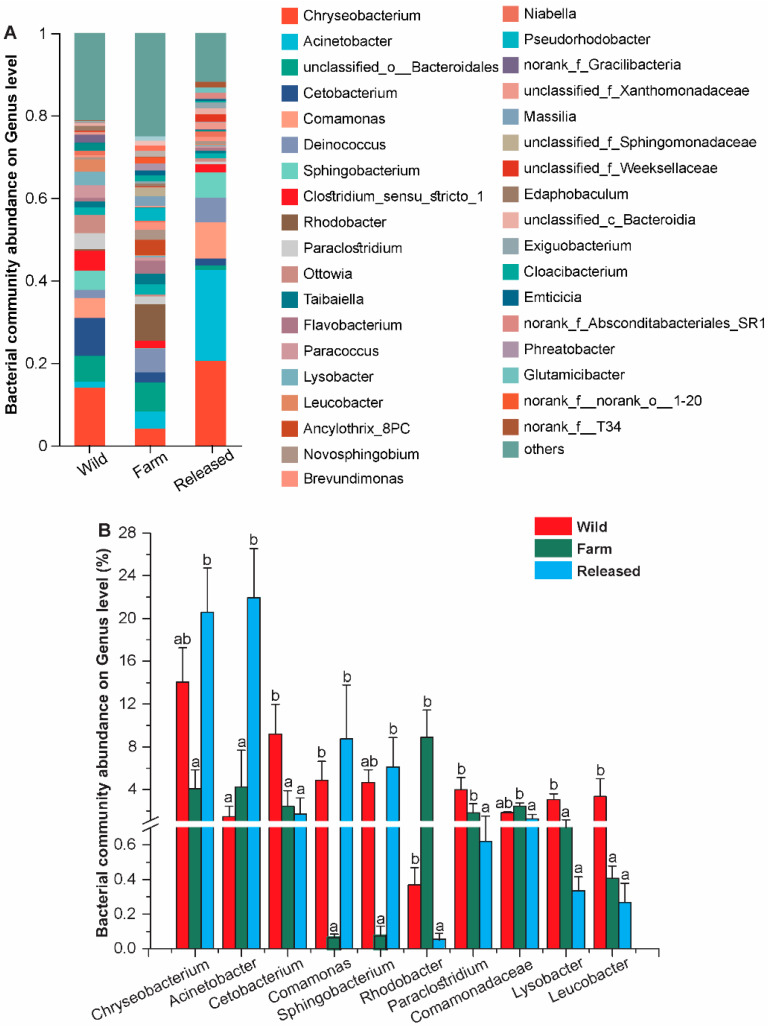
Comparison and relative abundances of intestinal bacterial genera in wild, farm, and released *M*. *reevesii*. (**A**) The most dominant genus. (**B**) The significant microbes at the genus level. The different lowercase letters represent significant differences between groups (*p* < 0.05).

At the genus level, the intestinal microbiota was dominated by *Chryseobacterium*, *Acinetobacter*, *Deinococcus*, *Comamonas*, and *Sphingobacterium*. Of them, *Chryseobacterium* (20.56%), *Acinetobacter* (21.93%), *Comamonas* (8.73%), and *Sphingobacterium* (6.13%) were abundant in the released group. In contrast, *Rhodobacter* (8.9%) and Comamonadaceae (2.45%) were significantly more abundant in the farm group (*p* < 0.05). In addition, *Cetobacterium* (9.20%), *Paraclostridium* (3.97%), *Lysobacter* (3.07%), and *Leucobacter* (3.33%) showed a greater abundance in the wild group than the farm and released groups (Figure 3).

### 3.2. Metagenomics Analysis

A total of nine fecal samples of *M. reevesii* from three different habitats (wild: *n* = 3; farm: *n* = 3; and released: *n* = 3) were analyzed through metagenomics sequences. After filtering, 416,281,918 reads were obtained and assembled, and 4,057,730 contigs (>500 pb) were obtained. A total of 5,863,311 genes were predicted with unigenes in the wild (1,654,026 ± 26,464.5), farm (2,075,938 ± 53,947.5), and released (2,133,347 ± 60,275.5) groups (Supplementary Spreadsheet S1). Moreover, 19,770 genes were shared in all three groups, while the number of unique genes in the wild, farm, and released groups was 1443, 1819, and 2782, respectively (Figure S3).

### 3.3. KEGG Pathway Analysis

**Figure 4:**
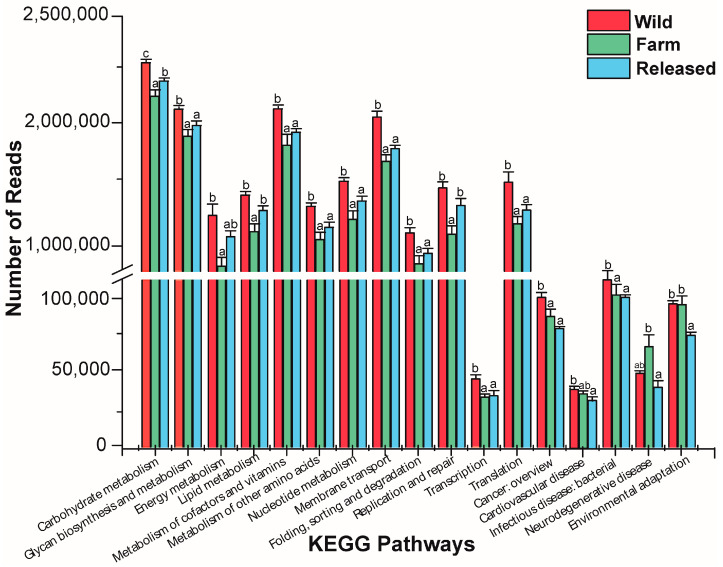
KEGG pathways are ample or depleted in the intestinal microbiota of wild, farm, and released *M. reevesii*. Different lowercase letters represent significant differences between groups.

Based on the KEGG database, the functions of the intestinal microbiota of the wild, farm, and released *M. reevesii* were examined. The KEGG level 1 pathway, in descending order, was metabolism, environmental information processing, genetic information processing, cellular processes, human diseases, and organismal systems. The level 2 KEGG pathways were almost significant among all three groups, as shown in Figure S4. Furthermore, in comparison of the wild with farm and released *M. reevesii*, the relative abundances of most pathways were significantly higher in the wild turtles, followed by the released and farm turtles, while neurodegenerative disease was only highly expressed in the farm turtles, as shown in Figure 4.

### 3.4. Antibiotic-Resistant Gene Analysis

**Figure 5:**
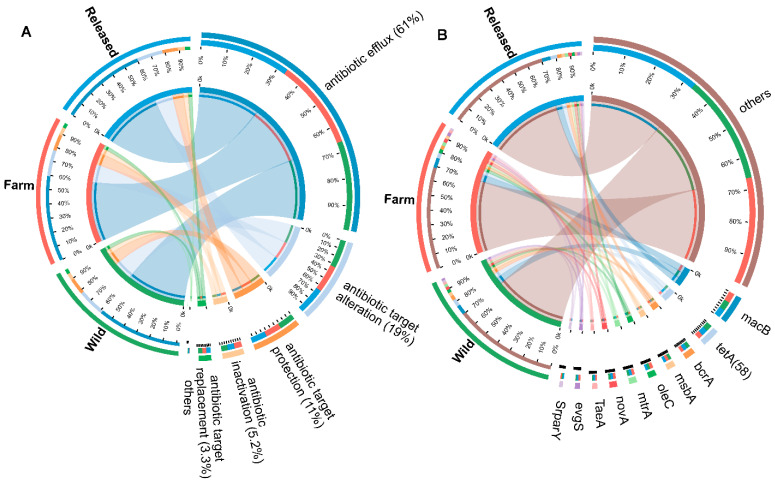
Resistance mechanisms and antibiotic resistance ontologies of antibiotic resistance genes. (**A**) Composition and proportion of resistance mechanisms in wild, farm, and released *M. reevesii*. (**B**) Functional distribution of each group’s top ten most abundant resistance ontologies. The colors green, red, and sky blue represent turtles from different habitats, while the other remaining colors show the percentage of resistance mechanisms in (**A**) and the proportion of selected ARGs among the groups in (**B**).

The predicted gene sequences were annotated based on the CARD database with a parameter setting of an e value < 1 × 10^30^ to investigate how different habitats influence the maintenance and spread of antibiotic-resistant genes. A total of 31,662 ARGs were detected in all the groups. Each gene was annotated with information on its resistance mechanisms and drug classes (Supplementary Spreadsheet S2). The main detected drug-resistant genes included antibiotic efflux (61%), antibiotic target alteration (19%), antibiotic target protection (11%), antibiotic inactivation (5.2%), antibiotic target replacement (3.3%), and others (0.29%) (Figure 5A). All ARGs were grouped into antibiotic resistance ontologies (AROs) (Supplementary Spreadsheet S3). The top 10 most abundant AROs are shown in Figure 5B. The genes *macB*, *oleC*, *mtrA*, *novA*, *TaeA*, and *evgS* were more abundant in the farm group than the released and wild groups, while *tetA*(58), *bcrA*, and *msbA* were higher in the wild group as compared to the released and farm groups. The *srpYmcr* gene only increased in the released group (Table 2).

**Figure 6:**
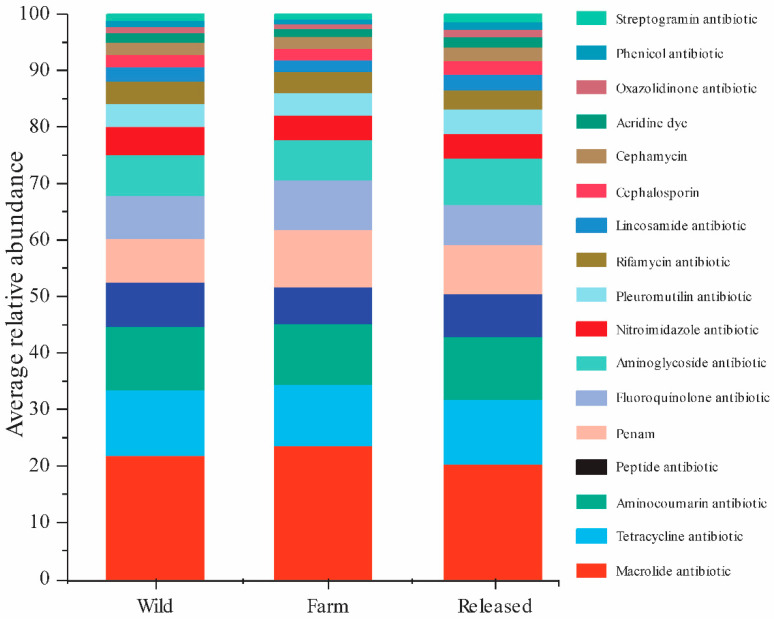
The average relative abundance of drug categories with antibiotic resistance genes in wild, farm, and released *M. reevesii*.

The relevant antibiotics and ARG types were matched in terms of the relative abundances of the genes responsible for antibiotic resistance in *M. reevesii* with various habitats, as summarized in Figure 6. The ARGs from all the samples showed resistance to 17 types of antibiotics that were found in the intestinal microbes. The top seven drug categories with ARG abundance in all the samples comprised macrolide, aminocoumarin, tetracycline, peptide, nitroimidazole, rifamycin, and acridine dye, whereas resistance to the remaining antibiotic classes was relatively low (Supplementary Spreadsheet S4). The relative abundances of these seven drug categories were compared in Table 3. It confirmed that all seven drug categories were significantly higher in the wild than the released and farm *M. reevesii*. Consequently, these results indicated a significant difference in ARG abundance in the wild, farm, and released *M. reevesii*.

## 4. Discussion

Previous studies have explored microorganisms present in the intestinal tract that strongly influence host health [32,54]. These gut microbiotas play a key role in host development, physiological functioning, health, and adaptation, which are greatly influenced by habitat and diet. A few studies on fish and rodents have highlighted the significant influence of habitat on the intestinal microbiota. For instance, studies on cavefish and Tibetan macaques demonstrate that habitat-specific microbes influence the gut microbiota [55,56]. Similarly, research on rodents in different habitat types shows that habitat succession stages significantly affect gut microbiota diversity and composition, emphasizing the role of habitats in shaping the gut microbiome of animals [57]. Likely, the external environment influences the composition and functions of the intestinal microbiota in *M. reevesii*, which needs to be further studied. To date, various studies have shed light on the intestinal microbiota of different living organisms, including turtles, by using 16S rRNA gene sequencing and metagenomics analysis [20,36]. However, this study is the first to use metagenomic sequencing analysis to examine the structure and functions of the intestinal microbiota in wild, farm, and released *M. reevesii*. Furthermore, this study provides insights into intestinal microbial diversity and composition, tracks habitat sources, explores KEGG pathways, and identifies ARGs in different groups of *M. reevesii.*

After the characterization of intestinal microbial communities in the fecal samples of *M. reevesii*, the alpha diversity and richness estimators confirmed significant differences (*p* < 0.05) in the wild, farm, and released groups. In contrast, the evenness estimators showed a significant difference between the farm and released turtles. In one study, geckos in a captive environment were exposed to human contact, which altered microbial diversity in their intestinal tracts [33]. Therefore, we speculate that farm turtles have a greater opportunity to interact with their keepers because microbiota acquired from hosts can be transmitted, which could colonize them. In addition, unique OTUs were also observed in all three groups, as shown in Figure S2. These unique OTUs are remarkable footprints for the habitat tracking of turtles, as studied in the wild and farmed yellow croakers [26]. Furthermore, it was confirmed that farm turtles maintain greater differences in the unweighted UniFrac distance, indicating that the intestinal microbiota structure of farm turtles shows no overlap with that of wild and released turtles. At the same time, they have a more significant divergence in intestinal microbiota when compared with other groups. Based on the PCoA and ANOSIM/Adonist results, the intestinal bacteria in wild, farm, and released *M. reevesii* had distinctly different bacterial communities. Earlier studies have also described fluctuations in bacterial communities in different vertebrates, including mammals [58], birds [59], reptiles [33], and fish [60]. In addition, it has been reported that the most dominant bacterial phyla in the intestines of other turtle species were Firmicutes, Bacteroida, Proteobacteria, and Desulfobacteriodata [61,62,63]. A dissimilarity in the relative proportion of a bacterial community can clarify a dietary and habitual change. The current study investigated the taxonomic composition of the gut microbiota among wild, farm, and released *M. reevesii*. The dominant phylum in the wild turtles was Bacteroidota, followed by Firmicutes, Fusobacteriota, and Actinobacteriota. In addition, Bacteroidota and Firmicutes were two dominant phyla, and this finding is consistent with studies on other turtle species, such as the gopher tortoise [64], the loggerhead sea turtle [65], and the painted turtle [66]. Furthermore, several studies confirmed that Bacteroidota and Firmicutes play a crucial role in energy metabolism and fulfill the energy requirements of the host [61,67]. Bacteriodota degrades polymeric organic compounds to digest food, encouraging energy-generating processes, while Firmicutes is involved in metabolizing plant polysaccharides and degrading cellulose into volatile fatty acids [20,61]. *M. reevesii* are omnivorous by nature; their diet includes a variety of small animals and leafy vegetables in the wild. While under farming conditions, these individuals feed on small fish, melons, and fruits (reptilesmagazine.com). In addition, it has been reported that carnivorous and omnivorous turtles’ intestinal microbiota is enriched by Bacteriodota [68]. Moreover, there is a mutualistic relation between the host and Bacteroidota that activates a T-cell-mediated response and limits the colonization of pathogens, thereby maintaining the individual’s health [69]. It has been stated that an increase in the relative abundance of Bacteroidota helps maintain the health of green turtles [63]. This suggests that the abundance of Bacteriodota in turtle species might help them digest food and resist disease. The intestines of various aquatic organisms also harbor Fusobacteria, which ferment carbohydrates and amino acids by producing butyrate [70]. Moreover, there are modulatory and anti-inflammatory properties due to butyric acid production associated with Fusobacteria [71]. In digestive systems, chlorofexi contains chemoorganoheterotrophic bacteria along with fermentative metabolisms. Therefore, all these phyla have been investigated before and may contribute to energy metabolism, vitamin synthesis, and immune responses.

At the genus level, we detected the following genera in all three groups of *M. reevessi* (3A/3B): *Chryseobacterium*, *Acinetobacter*, *Cetobacterium*, *Comamonas*, *Sphingobacterium*, *Rhodobacter*, *Paraclostridium*, Comamonadaceae, *Lysobacter*, and *Leucobacter*. *Chryseobacterium* is abundantly found in different environments, such as soil, aquatic environments, and animal intestines [72,73]. It has been reported that several species of *Chryseobacterium* are resistant to antibiotics and pathogenic to humans and aquatic animals [74,75]. Our results are also in line with those of Ilardi et al. (2010), who isolated Chryseobactirum from fish and aquatic habitats to show its antibiotic resistance and that it can be considered an opportunistic pathogen [76,77]. In the present study, *Acinetobacter* was more abundant in the released turtles compared to the farm turtles. Our results indicate that there might be limited chitin and lipid sources in farm diets compared to the released turtles’ environment. Moreover, it has been reported that some *Acinetobacter* species carry ARGs, while others are responsible for producing different enzymes, including chitinase or lipase, to help host digestion [78,79,80]. Likewise, *Cetobacterium* is a genus of Fusobacteria found in animal intestines. It was examined that *Cetobacterium* can produce vitamin B12, and increasing acetate production further helps in glucose homeostasis and carbohydrate utilization [81,82]. The increased levels of *Cetobacterium* might suggest that wild Chinese three-keeled pond turtles are deficient in vitamin B12. Furthermore, *Comamonas* and *Sphingobacterium* were also significantly abundant in the released group. In contrast, *Cetobacterium*, *Paraclostridium*, *Lysobacter*, and *Leucobacter* were most abundant in the wild group, followed by the farm and released groups (Figure 3A,B). According to all the above findings, diet and habitat play a pivotal role in influencing the intestinal microbiota of wild, farm, and released *M. reevessi*. They may also influence metabolic and other pathways.

The KEGG pathway comparison analysis exposed that many genes are involved in metabolism, genetic information processing, cellular processes, human diseases, and organismal systems in category 1 in *M. reevesii* among the three habitats. While in category 2, most pathways are related to metabolism, including carbohydrate metabolism, energy metabolism, the metabolism of cofactors and vitamins, nucleotide metabolism, lipid metabolism, and the metabolism of other amino acids. Glycan biosynthesis and metabolism were significantly abundant in the wild *M. reevesii* (Figure 4). The KEGG analysis revealed that habitat has substantial effects on metabolism and the role of food availability and intake on the intestinal microbiota in turtles. In addition, the KEGG pathway analysis revealed variations in the number of genes associated with different habitats, which is consistent with prior findings [36]. Furthermore, several reptile species, including the timber rattlesnake, the crocodile lizard, and the northern grass lizard, have been reported to possess genes involved in metabolism [21]. The same results have also been observed for birds [83] and mammals [84]. Therefore, we assumed that the metabolic variability of *M. reevesii* may have been impacted by the diverse habitats and the varying availability of resources. The variation in habitats and resulting alterations in food composition, stemming from the intricate nature of the ecosystem, perhaps led to a decline in natural resources [85]. This decline, in turn, may have had an impact on the growth of microbes inside the digestive tract of *M. reevesii*.

ARGs are environmental pollutants that pose potential risks to human and animal health due to the release of resistant pathogens, and they vary around the globe [86]. Many studies have stated that ARGs are found in various environmental compartments, such as fecal contents, soil, river water, and drinking water [41]. The present study found the most common and leading ARGs to be macrolides, aminocoumarin, and tetracycline resistance genes in each fecal content group. To compete with amino acids for ribosomal binding sites, macrolides inhibit protein synthesis [87]. In the present study, the macrolide ARG *macB* was most abundant in the farm (6.9%) group, followed by the wild (6.7%) and released (6.1%) groups. Our results align with previous studies showing that *macB* is more abundant in green sea turtles compared to hawksbill communities [41]. Moreover, Seribelli et al. (2021) also detected *macB* among the bacterial strains isolated from humans, food, and swine [88]. Tetracycline inhibits bacterial growth by inhibiting translation, and there are extensive uses for tetracycline in agriculture, veterinary medicine, and human therapy. Moreover, tetracycline antibiotics are also found in both terrestrial and aquatic environments and exert pressure on bacteria; as a result, ARGs are found in the environment [89]. Chen et al. (2022) stated that there is a great richness of tetracycline resistance genes in hawksbills and green sea turtles [41]. In the current study, tetracycline ARGs are also abundant in the wild group, which might be due to the discharge of wastewater from farms or via their prey, such as small wild animals with diverse habitat ranges. Founou et al. (2016) reported that food chains or direct contact are possible transmission routes for antibiotic-resistant microbes [90]. In addition, several studies have described that aquatic organism practices like fish farming confer antibiotic residues from various sources, including animal husbandry, permitting a pool of ARGs in the aquatic environment [91]. In the current study, *tetA(58*) showed the highest abundance in the wild group (5%), followed by the released (4.7%) and farm (3.7%) turtles. Aminocoumarins are antibiotics that inhibit DNA gyrase and have potential for use in drug development [92,93]. It was shown that novobiocin inhibited bacterial topoisomerase, specifically DNA gyrase, via its mode of action [94,95]. Moreover, it was confirmed that novobiocin is a potent inhibitor of the B-subunit of DNA gyrase and can inhibit DNA supercoiling [96]. Like other ARGs, *novA* is identified as a gene that is resistant to novobiocin. Previous studies have demonstrated that in *Bacillus subtilis*, novA mutations confer resistance to novobiocin [97,98]. In this study, novA was more abundant in the farm turtles (2.5%) as compared to the released (2.3%) and wild (2.1%) groups. In addition, some other antibiotics (peptides, Nitroimidazole, and Rifamycin) are also used for the treatment of various pathogenic bacterial infections. To promote yield in turtle farming and livestock, antibiotics are overused as feed additives or veterinary medicines. Therefore, turtle farming might be a potential reservoir for ARG pollution. Due to the overuse of these antibiotics, pathogenic bacteria become resistant due to ARGs. Further studies are needed to investigate the relationship between drug resistance (antimicrobial) and wild *M*. *reevesii*.

## 5. Conclusions

In conclusion, wild *M. revessii* showed higher microbial diversity than the farm and released turtles, while three unique OTUs belonged to the genera *Cellulosilyticum*, *Camelimonas*, and *norank_f_Alcaligenaceae* (unclassified) in the wild group, and one genus, *Deinococcusin*, in the released group happened to be a distinguishing biomarker in the gut microbiome as compared to the farm turtles. Moreover, Bacteriodota, Firmicutes, Fusobacteria, and Actinobacteria were more abundant in the wild group, but Chloroflexi showed greater abundance in the farm and released groups. In addition, *Cetobacterium*, *Paraclostridium*, *Lysobacter*, and *Leucobacter* were higher in the wild group than the farm and released groups, which is due to the various kinds of diets in the different habitats. Moreover, the relative abundances of most pathways were significantly higher in the wild turtles, followed by the released and farm turtles. The CARD database describes that the ARGs *macB*, *oleC*, *mtrA*, *novA*, *TaeA*, and *evgS* were the most abundant in the farm group, *tetA(58)*, *bcrA*, and *msbA* were higher in the wild group, and *srpYmcr* exhibited abundance only in the released group. ARGs in turtles can be attributed to antibiotic exposure in their habitats, like contaminated water or environments close to human settlements. Our findings will assist in the understanding of the relationship between the intestinal microbiota and its habitats, as well as provide knowledge about ARGs in *M. reevesii*. However, further studies are required to help protect, manage, and conserve this endangered species.

## Figures and Tables

**Table 1:** Intestinal bacterial diversity and richness estimators in wild, farm, and released *M. reevesii*. The values to the right of the ± represent the standard error mean. Different lowercase letters represent significant differences between groups.

Habitats	Richness and Diversity Estimators				
	OTUs	ACE	Chao1	Shannon	Simpson
**Wild**	247.50 ± 14.34 ^a^	337.84 ± 13.7 ^a^	353.87 ± 16.65 ^a^	3.7504 ± 0.16 ^a^	0.05 ± 0.00 ^ab^
**Farm**	408.80 ± 5.82 ^b^	539.06 ± 7.53 ^c^	559.51 ± 7.53 ^b^	4.2813 ± 0.08 ^b^	0.03 ± 0.00 ^a^
**Released**	282.60 ± 9.57 ^a^	379.93 ± 13.98 ^b^	393.80 ± 18.38 ^a^	3.5435 ± 0.19 ^a^	0.07 ± 0.01 ^b^

**Table 2:** Antibiotic resistance genes in the intestinal microbiota of wild, farm, and released *M. reevesii*. The different lowercase letters represent significant differences between groups, and the letters with no superscript represent values that are not significantly different from each other. The values to the right of ± represent the standard error mean.

ARGs	Wild	Farm	Released
*macB*	6.65 ± 0.21 ^ab^	6.94 ± 0.17 ^b^	6.08 ± 0.28 ^a^
*tetA(58)*	5.01 ± 0.24 ^b^	3.74 ± 0.09 ^a^	4.65 ± 0.52 ^ab^
*bcrA*	3.14 ± 0.11	2.73 ± 0.04	3.03 ± 0.25
*msbA*	2.63 ± 0.12	2.48 ± 0.08	2.46 ± 0.19
*oleC*	2.29 ± 0.19	2.76 ± 0.09	2.14 ± 0.28
*mtrA*	2.01 ± 0.13	2.61 ± 0.18	2.32 ± 0.28
*novA*	2.06 ± 0.30	2.51 ± 0.14	2.31 ± 0.23
*TaeA*	1.60 ± 0.01 ^a^	1.91 ± 0.03 ^b^	1.82 ± 0.03 ^b^
*evgS*	1.31 ± 0.12 ^a^	2.38 ± 0.08 ^b^	1.62 ± 0.20 ^a^
*srpYmcr*	1.47 ± 0.05	1.43 ± 0.06	1.55 ± 0.08
Others	71.80 ± 0.91	70.47 ± 0.66	71.96 ± 1.08

**Table 3:** The drug categories with antibiotic resistance gene abundance comparison. The different lowercase letters represent significant differences among wild, farm, and released *M. reevesii*.

Class	Wild	Farm	Released
Macrolide antibiotic	407,268 ± 10,238 ^b^	319,132 ± 11,347 ^a^	320,378 ± 6298 ^a^
Aminocoumarin antibiotic	209,156 ± 7231 ^b^	144,748 ± 8112 ^a^	173,676 ± 4845 ^ab^
Tetracycline antibiotic	217,246 ± 7573 ^b^	146,014 ± 6704 ^a^	180,496 ± 5449 ^ab^
Peptide antibiotic	145,318 ± 2303 ^c^	88,592 ± 397 ^a^	120,184 ± 2470 ^b^
Nitroimidazole antibiotic	92,770 ± 2720 ^b^	58,586 ± 2643 ^a^	68,382 ± 1379 ^a^
Rifamycin antibiotic	73,508 ± 2612 ^b^	51,152 ± 19,550 ^a^	53,920 ± 1805 ^a^
Acridine dye	32,406 ± 2425 ^b^	18,526 ± 9649 ^a^	27,502 ± 1128 ^ab^

## Data Availability

The raw 16S rRNA gene sequencing is available in the NCBI Sequence Read Archive under the accession number PRJNA994721, and the metagenome sequencing data are available under the accession number PRJNA1028484.

## Supplementary Materials

The following supporting information can be downloaded at https://www.mdpi.com/article/10.3390/ani14121750/s1, Figure S1: Rarefaction curve analysis of the observed species per sample of *M. reevissi*; Figure S2: Venn diagram indicates the number of unique and shared OTUs in wild, farm, and released *M. reevissi*; Figure S3: Venn diagram indicates the number of unique and shared genes in wild, farm, and released *M. reevissi*; Figure S4: KEGG pathways in intestinal microbiota of wild, farm, and released *M. reevesii*. * Represent the significant difference among groups (*p* < 0.05); Spreadsheet S1: Total raw reads, clean reads, contigs, and total genes in wild, farm, and released *M*. *reevesii*; Spreadsheet S2: Genes information for resistance mechanisms and drug classes; Spreadsheet S3: Antibiotic resistance ontologies; Spreadsheet S4: ARGs showed resistance to different drug classes.
