# Supplementary material for: Metagenomics Analysis Reveals the Composition and Functional Differences of Fecal Microbiota in Wild, Farm, and Released Chinese Three-Keeled Pond Turtles (Mauremys reevesii)
Source: Animals (Basel). 2024 Jun 10;14(12):1750. doi: 10.3390/ani14121750 (PMC11201187; doi:10.3390/ani14121750)
Supplement: Supplementary file 1 [file animals-14-01750-s001.zip › animals-3002031-supplementary/Supplymentry Data/Supplementary Figures.doc]

**Supplementary Figure S1.** Rarefaction curve analysis of the observed species per sample of *M*. *reevissi*.

**Supplementary Figure S2.** Venn diagram indicates the number of unique and shared OTUs in wild, farm, and released *M*. *reevissi*.

**Supplementary Figure S3.** Venn diagram indicates the number of unique and shared genes in wild, farm, and released *M*. *reevissi*.

**Supplementary Figure S4.** KEGG pathways in intestinal microbiota of wild, farm, and released *M. reevesii*. * Represent the significant difference among groups (*P* *<* 0.05).
